# Universal versus conditional day 3 follow-up for children with non-severe unclassified fever at the community level in Ethiopia: A cluster-randomised non-inferiority trial

**DOI:** 10.1371/journal.pmed.1002553

**Published:** 2018-04-17

**Authors:** Karin Källander, Tobias Alfvén, Tjede Funk, Ayalkibet Abebe, Abreham Hailemariam, Dawit Getachew, Max Petzold, Laura C. Steinhardt, Julie R. Gutman

**Affiliations:** 1 Malaria Consortium, London, United Kingdom; 2 Department of Public Health Sciences, Karolinska Institutet, Stockholm, Sweden; 3 Sachs’ Children and Youth Hospital, Stockholm, Sweden; 4 Malaria Consortium, Addis Ababa, Ethiopia; 5 Health Metrics, Sahlgrenska Academy, University of Gothenburg, Gothenburg, Sweden; 6 School of Public Health, Faculty of Health Sciences, University of the Witwatersrand, Johannesburg, South Africa; 7 Malaria Branch, Division of Parasitic Diseases and Malaria, Center for Global Health, US Centers for Disease Control and Prevention, Atlanta, Georgia, United States of America; London School of Hygiene & Tropical Medicine, UNITED KINGDOM

## Abstract

**Background:**

With declining malaria prevalence and improved use of malaria diagnostic tests, an increasing proportion of children seen by community health workers (CHWs) have unclassified fever. Current community management guidelines by WHO advise that children seen with non-severe unclassified fever (on day 1) should return to CHWs on day 3 for reassessment. We compared the safety of conditional follow-up reassessment only in cases where symptoms do not resolve with universal follow-up on day 3.

**Methods and findings:**

We undertook a 2-arm cluster-randomised controlled non-inferiority trial among children aged 2–59 months presenting with fever and without malaria, pneumonia, diarrhoea, or danger signs to 284 CHWs affiliated with 25 health centres (clusters) in Southern Nations, Nationalities, and Peoples’ Region, Ethiopia. The primary outcome was treatment failure (persistent fever, development of danger signs, hospital admission, death, malaria, pneumonia, or diarrhoea) at 1 week (day 8) of follow-up. Non-inferiority was defined as a 4% or smaller difference in the proportion of treatment failures with conditional follow-up compared to universal follow-up. Secondary outcomes included the percentage of children brought for reassessment, antimicrobial prescription, and severe adverse events (hospitalisations and deaths) after 4 weeks (day 29). From December 1, 2015, to November 30, 2016, we enrolled 4,595 children, of whom 3,946 (1,953 universal follow-up arm; 1,993 conditional follow-up arm) adhered to the CHW’s follow-up advice and also completed a day 8 study visit within ±1 days. Overall, 2.7% had treatment failure on day 8: 0.8% (16/1,993) in the conditional follow-up arm and 4.6% (90/1,953) in the universal follow-up arm (risk difference of treatment failure −3.81%, 95% CI −∞, 0.65%), meeting the prespecified criterion for non-inferiority. There were no deaths recorded by day 29. In the universal follow-up arm, 94.6% of caregivers reported returning for reassessment on day 3, in contrast to 7.5% in the conditional follow-up arm (risk ratio 22.0, 95% CI 17.9, 27.2, *p* < 0.001). Few children sought care from another provider after their initial visit to the CHW: 3.0% (59/1,993) in the conditional follow-up arm and 1.1% (22/1,953) in the universal follow-up arm, on average 3.2 and 3.4 days later, respectively, with no significant difference between arms (risk difference 1.79%, 95% CI −1.23%, 4.82%, *p* = 0.244). The mean travel time to another provider was 2.2 hours (95% CI 0.01, 5.3) in the conditional follow-up arm and 2.6 hours (95% CI 0.02, 4.5) in the universal follow-up arm (*p =* 0.82); the mean cost for seeking care after visiting the CHW was 26.5 birr (95% CI 7.8, 45.2) and 22.8 birr (95% CI 15.6, 30.0), respectively (*p =* 0.69). Though this study was an important step to evaluate the safety of conditional follow-up, the high adherence seen may have resulted from knowledge of the 1-week follow-up visit and may therefore not transfer to routine practice; hence, in an implementation setting it is crucial that CHWs are well trained in counselling skills to advise caregivers on when to come back for follow-up.

**Conclusions:**

Conditional follow-up of children with non-severe unclassified fever in a low malaria endemic setting in Ethiopia was non-inferior to universal follow-up through day 8. Allowing CHWs to advise caregivers to bring children back only in case of continued symptoms might be a more efficient use of resources in similar settings.

**Trial registration:**

www.clinicaltrials.gov, identifier NCT02926625

## Abstract 2

In a cluster-randomized trial conducted in Ethiopia, Karin Källander and colleagues provide evidence that conditional follow-up of children with non-severe unclassified fever in a low-malaria endemic setting can be non-inferior to universal follow-up.

## Author summary

**Why was this study done?:**

- As a result of declining malaria prevalence and increased use of malaria diagnostic tests, it is becoming more common for children seen by community health workers (CHWs) to have non-severe unclassified fever.
- Caregivers of children seen on day 1 with non-severe unclassified fever are advised to bring the child back to the CHW on day 3 for reassessment, regardless of whether symptoms have resolved or not (universal follow-up), burdening both the family and health system.
- This study assessed the safety of CHWs following-up children with non-severe unclassified fever only when symptoms have not resolved (conditional follow-up), hypothesizing that the conditional follow-up would be as safe as universal follow-up.

**What did the researchers do and find?:**

- From December 1, 2015, to November 30, 2016, a total of 4,179 children were enrolled into the study in the Southern Nations, Nationalities, and Peoples’ Region in Ethiopia, of whom 3,946 (1,953 universal follow-up arm; 1,993 conditional follow-up arm) adhered to the CHW’s follow-up advice and also completed a day 8 study visit within ±1 days.
- Caregivers’ adherence with follow-up advice given by CHWs was high: 93.5% of caregivers followed the advice that was given in line with the cluster to which the CHW was assigned.
- On day 8, a total of 2.7% of the children had treatment failure (persistent fever, development of danger signs, hospital admission, death, malaria, pneumonia, or diarrhoea), with no significant difference between the 2 follow-up arms.
- There were no deaths recorded by day 29.

**What do these findings mean?:**

- Conditional follow-up of children with non-severe unclassified fever in a low malaria endemic setting was as safe as universal follow-up.
- Allowing CHWs to advise caregivers to return only if a child has continued symptoms may be more efficient than their advising all children to return.

## Introduction

Mortality in children under 5 years is estimated at 43/1,000 live births globally and 82/1,000 live births in sub-Saharan Africa. This corresponds to the death of 5.6 million children under 5 years old globally each year, 2.8 million in sub-Saharan Africa alone [1].

Although there have been substantial improvements over the last 2 decades, high levels of child mortality persist in many countries, including Ethiopia, where the under-5 mortality rate is estimated at 58/1,000 live births [1,2]. A large proportion of deaths globally are caused by infectious diseases such as pneumonia (15.5%), diarrhoea (8.9%), and malaria (5.2%) [3]. In response, many countries in sub-Saharan Africa have introduced integrated community case management (iCCM), where community health workers (CHWs) are trained to assess, classify, and treat uncomplicated cases of pneumonia, diarrhoea, and malaria in children under 5 years, and refer children with danger signs and malnutrition for facility-based care [4]. While the mortality impact of iCCM has been difficult to demonstrate [5], there is clear evidence that it can increase the treatment rate among sick children [6].

As per WHO iCCM guidelines [7], children diagnosed by a CHW with a non-severe illness are given treatment and counselled to return on day 3 to assess treatment compliance and illness resolution. Children with fever but without a diagnosable cause of illness and without danger signs (i.e., unclassified fever) for whom anti-infective treatment can be withheld are also told to return to the CHW on day 3, even if the child has recovered.

However, febrile illness is common in childhood, and is often due to viruses or other self-resolving illnesses [8,9]. In a large proportion of cases, fever resolves rapidly, most often within 96 hours [10]. A number of studies have suggested that it is safe to withhold medical treatment for children with unclassified fever [11,12]. The Integrated Management of Neonatal and Childhood Illness (IMNCI) manual [13], followed by CHWs in Ethiopia (locally referred to as health extension workers [HEWs]), recommends that such children should return for a reassessment only if the illness persists or deteriorates.

There is limited evidence on which of the 2 follow-up recommendations (conditional, as in IMNCI, or universal, as in iCCM) is safer for the child. Further, it is unclear whether and to what extent caregivers of children actually come back promptly to CHWs for their conditional follow-up visit if the child is not improving, or if they come back at all if the child has improved. Bacterial infections can develop quickly, and delaying care-seeking is a major risk factor for death in both pneumonia and malaria [14,15]; hence, children with untreated persistent fever may be at risk if caregivers do not comply with the conditional follow-up advice. A universal follow-up visit for all children may promote detection of those at risk of developing severe illness. However, it could also potentially lead to delayed care-seeking for children who rapidly deteriorate at home if caregivers wait for their booked follow-up visit. In addition, the visit may add extra burden to families and CHWs, and might be unnecessary if fever has resolved [16,17]. On the caregiver side, opportunity costs and other barriers often hinder care-seeking for sick children, even when community-based providers are near and free of charge [18]. It is therefore unclear whether caregivers and CHWs would comply better with the conditional follow-up advice compared to the universal follow-up advice and whether the universal follow-up visit is even necessary.

Hypothesizing that conditional follow-up does not have a higher risk of treatment failure, the objective of this study was to assess whether conditional follow-up was non-inferior to universal follow-up for non-severe febrile illness in children aged 2 to 59 months in whom malaria, pneumonia, diarrhoea, and danger signs were absent.

## Methods

### Study design

This was a 2-arm community cluster-randomised non-inferiority trial (the TRAction study) carried out in 3 woredas (districts) in Southern Nations, Nationalities, and Peoples’ Region (SNNPR) in Ethiopia. The woredas were purposively selected based on (1) strength of iCCM programme (i.e., consistency in HEW supervision and supply), (2) HEW use rate among caregivers (more than 50 children assessed for fever each month over a 12-month period), and (3) regular concurrent community mobilisation and supportive supervision activities under other grants (to ensure that demand was kept high during the study period). Clusters, defined by health centre (the referral centre and practical training institution for HEWs, where their services are coordinated), were randomised into either the conditional or universal follow-up arm. All 25 health centres and 144 health posts with 284 HEWs in the 3 selected woredas were included in the study, and all children seeking care from the health posts in these clusters were potential recipients of the interventions, in addition to having access to routine care available from private and public health services. Caregivers of children who met the inclusion criteria (fever with a negative malaria rapid diagnostic test [mRDT], and in whom the HEW did not diagnose pneumonia or diarrhoea or identify other symptoms requiring referral on day 1) were counselled to either (1) return on day 3 (universal follow-up arm) or (2) return if symptoms persisted (conditional follow-up arm). Caregivers in both arms were advised to go to the health centre immediately if danger signs, such as convulsion, lethargy, not drinking/breastfeeding, or vomiting everything, developed. Of 25 included clusters, 13 were randomised to the universal follow-up arm and 12 to the conditional follow-up arm.

### Study setting

The government of Ethiopia has deployed over 42,000 female CHWs, or HEWs [19,20], to provide preventive, promotive, and curative health services at the community level; since 2010, the full iCCM package has been included in the IMNCI guidelines (with the addition of treatment of pneumonia) and has been scaled up in most regions of the country. There are typically 2 HEWs assigned to a health post in a sub-district with a population of 3,000–5,000; they are supported by the Health Development Army, female volunteers who enhance community engagement and encourage use of maternal and newborn health services [21]. While IMNCI recommends conditional follow-up, HEWs and their supervisors report a range of other practices for children with unclassified fever, including universal follow-up advice, immediate referral to health centres, and treatment with antimalarial tablets [22].

### Participants

Children aged 2–59 months who presented to the HEWs in the study area with fever (≥37.5°C) or a history of fever, a negative mRDT, no pneumonia or diarrhoea according to iCCM criteria, and no danger signs were eligible to participate in the study. Written informed consent was obtained from each caregiver before enrolment in the study.

### Randomisation and masking

Cluster randomisation was at the health centre area level; the 25 study health centres had an average of 5 health posts and 7.5 HEWs each. Allocation to the universal versus conditional arm was done via restricted randomisation whereby clusters were balanced on health area estimates of (1) population size, (2) prior 6-month likelihood of mRDT-negative febrile children (number of children mRDT negative/under-5 population), and (3) geographic distance from health post to zonal referral hospital, was performed to minimise the differences between conditional and universal follow-up arms [23]. Sorting of clusters and random selection of schemes were carried out by the study statistician (MP) in Stata 13 (StataCorp, College Station, TX, US).

### Procedures

HEWs collected data at enrolment (day 1) using an Open Data Kit (ODK) Collect (version 1.9.1) data collection form on mobile phones, including date of enrolment, a child identifier code, and clinical indicators such as fever (axillary temperature ≥ 37.5°C or, if a functional thermometer was unavailable, hot to touch reported by HEW or caregiver-reported fever in past 2 days), cough, respiratory rate, diarrhoea, and danger signs. The enrolment data were synchronised with a server accessed by a data manager for scheduling of follow-up visits. Six independent evaluators (IEs), with a bachelor’s degree in a health-related discipline, clinical experience using IMNCI, and a minimum of 2 years’ research experience, and who were able to communicate in Amharic and English, were trained for 2 days in study procedures and in follow-up of enrolled children. Each district was assigned 2 IEs, who were blinded to the cluster allocation of the children they were reassessing.

In the conditional follow-up arm, HEWs counselled caregivers on how to detect danger signs and to seek care immediately at the health centre if danger signs developed, how to reduce fever using paracetamol, and the need to return at any point to the HEW for reassessment if symptoms remained the same, or worsened. In the universal follow-up arm, caregivers were counselled on all of the above, as well as the need to return on day 3 to the HEW for a follow-up assessment, even if the child had recovered. Caregivers in both arms were informed that a follow-up home visit by an IE would take place. Clinical outcomes were assessed by an IE during a home visit after 1 week (on day 8); if the child had not fully recovered, the child was assessed again by the IE after 2 weeks (day 15) and, if still not recovered, at after 4 weeks (day 29). Caregivers of all children were followed up by a phone call to assess vital status (alive/dead) after 4 weeks. Management of illness at any follow-up visit (i.e., return to HEW on any day, including scheduled assessments) followed national IMNCI guidelines.

IEs initially used ODK to collect reassessment data on enrolled children; halfway through the study the data collection software was changed to CommCare (version 2.38.1, Dimagi, Cambridge, MA, US), which allowed for automatic linking of follow-up forms, as well as scheduling of subsequent visits, once the children were registered in the 1-week follow-up form. The replacement system used an automatically generated child identifier code, which reduced the effort of having to manually link the forms, as well as supporting the IEs in tracking the follow-up visits that were due. The data collected during the household follow-up visits included the child identifier code, clinical data, additional antimicrobial treatment, hospitalisation, care-seeking history, and costs, as well as caregiver and household characteristics.

For children who were brought back on day 3 for reassessment in the universal follow-up arm and for any spontaneous revisit in both arms, a full reassessment was done by the HEW. If the child still had unclassified fever and a negative mRDT on reassessment, the child was referred to the nearest health centre, as recommended in the national IMNCI guidelines.

A rigorous monitoring system implemented by the study team was part of the continuous quality assurance. The data manager reviewed forms submitted to the server daily, and checked for duplicates, completeness, and accuracy before storing them in the project database. Discrepancies, overdue follow-up visits, and other issues were resolved by phone calls to the IEs and during weekly supervision meetings with field research staff. Biweekly field supervision visits to all HEWs were carried out, and district HEW supervisors were trained to monitor HEW trial activities during routine weekly group supervisions. While the protocol stated that a minimum of 10% of all enrolled cases and 50% of children with treatment failure should have a quality control reassessment by a research assistant, the actual percentage was significantly higher. Six months into the trial, all HEWs and their district supervisors had a refresher training in study procedures. In addition, the regional ethical clearance committee members did a field supervision visit during implementation of the project in all 3 districts selected for the study (9 health posts; 3 from each district), and provided feedback recommendations to the study team. The final dataset was analysed in Stata 13.

### Outcomes

The primary outcome was treatment failure rate on day 8, defined as the proportion of children whose illness was not resolved (child had any of the following: reported fever, danger sign(s), hospital admission, death, malaria, pneumonia, or diarrhoea). Three progressively stricter, and more objective, definitions of treatment failure were added in post hoc analysis to be consistent with a concurrent sister study in the Democratic Republic of the Congo (DRC) [24]: (1) reported fever ≥3 days, danger sign, hospital admission, death, malaria, pneumonia, or diarrhoea; (2) measured axillary temperature ≥ 37.5°C, danger sign, hospital admission, death, malaria, pneumonia, or diarrhoea; and (3) danger sign, hospital admission, death, malaria, pneumonia, or diarrhoea.

Secondary outcomes were percentage of caregivers who brought the child to the HEW for the follow-up visit on day 3 in the universal follow-up arm; percentage of caregivers who spontaneously re-presented to HEW for persistence or worsening of symptoms in the conditional follow-up arm, and the timing of these visits; percentage of children receiving antimicrobial treatment in each arm; and severe adverse events in each arm. Severe adverse events were defined as hospitalisation or death. A data monitoring committee (DMC) convened twice during the study to review enrolment rates, demographic and clinical characteristics of enrolled children, and follow-up rates at 1, 2, and 4 weeks, in order to monitor the overall conduct of the study. The DMC was advisory to a study steering committee (SC), which comprised the implementing study team from Malaria Consortium and lead study investigators, who jointly had responsibility for the design, conduct, and analysis of the trial. The SC was responsible for reviewing the DMC recommendations, to decide whether to continue or terminate the study, and to determine whether amendments to the protocol or changes in study conduct were required.

### Statistical analysis

We hypothesized that treatment failure at day 8 would not be more common with conditional than universal follow-up. We assumed that about 5% of children in the universal follow-up arm and 6% in the conditional follow-up arm would have treatment failure at day 8 (based on rates of 3% and 8% in previous studies [9,25]). Sample size for a non-inferiority trial was calculated in PASS 15 (NCSS, Kaysville, UT, US). Assuming that the proportion of failure was 5% in the universal follow-up arm and 6% in the conditional follow-up arm, and allowing for non-inferiority if the proportion of failure was as high as 9% in the conditional follow-up arm, a sample size of 2,142 per arm was needed to ensure that the upper limit of the 1-sided 95% confidence interval would exclude a difference in treatment failure of more than 4% with power of 80%. Using a design effect of 3 to account for clustering at the health post and health centre levels, the total sample size required was 4,284 children, with 2,142 per arm; this was inflated to 4,900 to account for potential losses to follow-up. The primary analysis was conducted on the per-protocol population (only including children for whom the primary outcome was collected on day 8 ± 1 and whose caregiver reported receiving follow-up advice from the HEW that was aligned with the study arm). In addition, an intention-to-treat analysis was done, whereby all children with a primary outcome defined were included. We also calculated cluster-specific failure rates on the per-protocol population and with the same model specifications as for the primary outcome.

The primary outcome was compared between arms using generalised linear models with a binomial distribution and identity link using a robust variance estimator, treating cluster as a random effect. We applied a conventional statistical non-inferiority test using a CI approach using the exact binomial CI for the difference in overall treatment failure between study arms. Here, we claimed non-inferiority if the upper bound of the 95% CI lay on the negative side of the 4% margin, using a 1-sided test done at the 2.5% significance level. The main analysis was done using the per-protocol population, as is appropriate for non-inferiority and equivalence studies, together with sensitivity analysis in the per-protocol and intention-to-treat populations [26]. All *p*-values for categorical data were calculated using the Pearson’s chi-squared test, whereas the adjusted Wald test was used for continuous data, accounting for clustering using the *svy* command in Stata 13.

### Ethical approvals

The trial protocol was approved by the SNNPR State Health Bureau on September 23, 2015 (ref P026-19/4511). In addition, approval was obtained from the district authorities and local leaders in the study-area woredas. US Centers for Disease Control and Prevention investigators participated under a non-engaged determination from their Office for Human Research Protections. The study protocol has been published [23] and was registered (ClinicalTrials.gov; identifier NCT02926625) after the first participant was randomised due to a miscommunication between study investigators.

## Results

**Fig 1:**
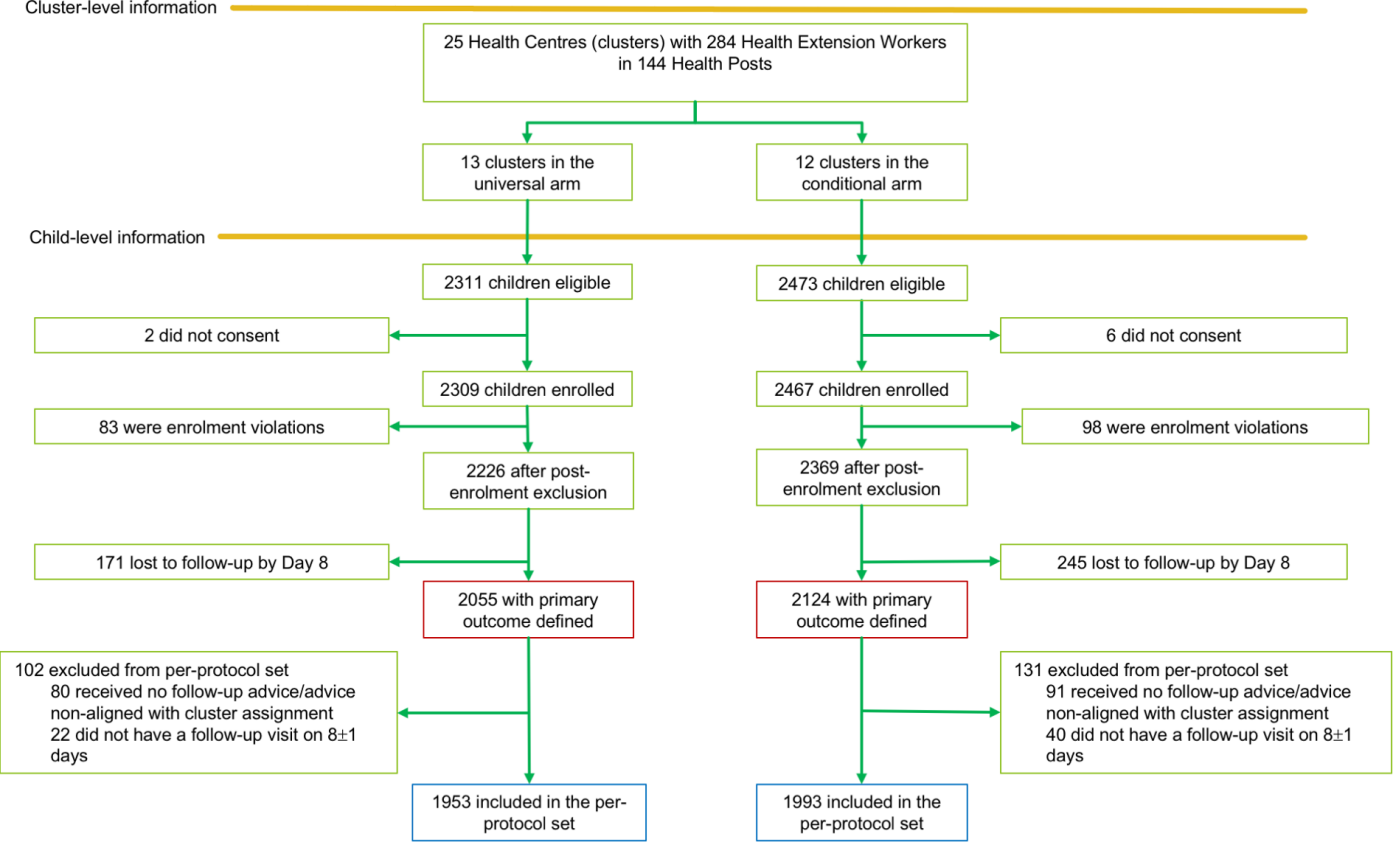
Trial profile of the TRAction Ethiopia study.

From December 1, 2015, to November 30, 2016, 4,784 children were eligible for enrolment; consent for enrolment was not obtained for 8 of these. In all, 4,776 children were enrolled (mean 191 per cluster [range 45–762]), but 181 were excluded due to enrolment violations (fever not reported/measured, presence of diarrhoea or pneumonia at enrolment, or outside the eligible age group) (Fig 1). The mean number of children enrolled per HEW was 20.8 (range 1–103) in the universal follow-up arm and 22.1 (range 1–166) in the conditional follow-up arm.

Baseline characteristics were well balanced between arms (Table 1). There were more children with cough in the conditional follow-up arm than in the universal follow-up arm (*p* < 0.001).

**Table 1:** Baseline characteristics of children enrolled.

Characteristic	Arm			
	Conditional		Universal	
	*N* or mean	Percent or range	*N* or mean	Percent or range
Sex				
Male	1,216	51.3%	1,190	53.5%
Female	1,153	48.7%	1,036	46.5%
Age (months)				
2–11	679	28.7%	542	24.3%
12–23	628	26.5%	673	30.2%
24–35	407	17.2%	426	19.1%
36–47	393	16.6%	373	16.8%
48–59	262	11.1%	212	9.5%
Cough	340	14.4%	206	9.3%
Temperature measured	2,343	98.9%	2,190	98.4%
Axillary temperature ≥ 37.5°C	1,024	43.2%	1,041	46.8%
Hot to HEW touch	1,122	47.4%	1,018	45.7%
Reported fever	223	9.4%	167	7.5%
Axillary temperature (degrees Celsius)	37.1	33.6–40.7	37.2	33.4–40
Weight (kilograms)	9.3	2.0–23.0	10.0	1.5–21.7

HEW, health extension worker.

Follow-up was completed in December 2016, 1 month after the last patient was enrolled. Of the 4,595 children enrolled, 416 were lost to follow-up by day 8, resulting in a total of 4,179 children with the primary outcome defined, including 3,946 who met the per-protocol definition (Fig 1). Late follow-up and loss to follow-up were primarily due to difficulties in accessing the villages during the rainy season, as well as the close-down of the mobile data network (and hence inability of HEWs to send data on enrolled cases) during a state of emergency that was instituted by the Ethiopian government in October 2016.

In all, 97.3% (4,064/4,179) of caregivers reported receiving follow-up advice, and about 97% received advice that was in line with the cluster allocation (Table 2). Caregivers’ reported adherence with the advice given by the HEWs was high: 94.6% of caregivers in the universal follow-up arm reported returning to the HEW, in contrast to only 7.5% in the conditional follow-up arm (risk ratio 22.0, 95% CI 17.9, 27.2).

**Table 2:** Caregivers’ reported receipt of advice and adherence with advice given by HEW.

Outcome	Arm			
	Conditional		Universal	
	*N*	Percent	*N*	Percent
Any follow-up advice given by HEW	2,047	98.2	2,017	98.2
Follow-up advice given in line with cluster allocation	1,992	97.3	1,971	97.7
Returned to the HEW	153	7.5	1,907	94.6

HEW, health extension worker.

Overall, 106 (2.7%) of the 3,946 enrolled children had treatment failure at day 8: 0.8% (16/1,993) in the conditional follow-up arm and 4.6% (90/1,953) in the universal follow-up arm. The treatment failure rate varied by cluster and ranged from 0% to 12% (intraclass correlation coefficient 0.07), and the total number of enrolments was proportionate to cluster size for all but 3 clusters (Table 3).

**Table 3:** Treatment failure rate at 1 week among children enrolled, by arm and cluster.

Arm	Cluster number	Treatment failure		Total enrolled	Percent
		No	Yes		
**Conditional**	3	80	5	85	5.9
	4	114	3	117	2.6
	5	197	0	197	0.0
	7	267	4	271	1.5
	9	151	0	151	0.0
	10	157	3	160	1.9
	11	488	0	488	0.0
	13	32	0	32	0.0
	14	85	1	86	1.2
	15	79	0	79	0.0
	23	118	0	118	0.0
	24	209	0	209	0.0
**Universal**	1	34	0	34	0.0
	2	120	0	120	0.0
	6	110	1	111	0.9
	8	573	78	651	12.0
	12	80	0	80	0.0
	16	69	2	71	2.8
	17	94	1	94	1.1
	18	43	0	43	0.0
	19	324	0	324	0.0
	20	140	4	144	2.8
	21	77	0	77	0.0
	22	94	1	95	1.1
	25	105	3	108	2.8

**Fig 2:**
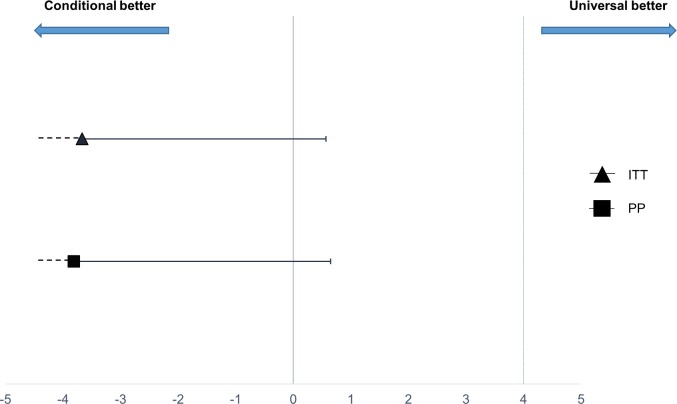
Non-inferiority plot comparing treatment failure outcome at 1 week in the conditional and universal follow-up arms. The point estimates of the risk difference in treatment failure at day 8 and their respective upper 95% confidence intervals are displayed in black. The dotted vertical line shows the predefined inferiority margin of 4%. In none of the performed analyses was the non-inferiority margin exceeded. ITT, intention-to-treat; PP, per-protocol.

The difference in treatment failure between conditional follow-up and universal follow-up was −3.81% (95% CI −∞, 0.65%) and −3.67% (95% CI −∞, 0.57%) in the per-protocol and the intention-to-treat populations, respectively (Table 4). As the difference between arms was less than the prespecified acceptable margin of 4% for the upper 95% CI, conditional follow-up was non-inferior to universal follow-up. Applying the more stringent treatment failure definitions did not change the result and further reduced the difference between the 2 arms (Table 4). The non-inferiority plot of clinical failure in the intention-to-treat and per-protocol populations is displayed in Fig 2.

**Table 4:** Comparison of the primary outcome, treatment failure at 1 week, between treatment arms.

Primary outcome definition	Arm		Difference	Upper limit 95% CI	*p*-Value
	Conditional, *n* (percent)	Universal, *n* (percent)			
**Treatment failure 1 (danger sign, hospital admission, death, malaria, pneumonia, diarrhoea, or reported fever)**					
Per-protocol	16 (0.80)	90 (4.61)	−3.81%	0.65	0.002
Intention-to-treat	19 (0.89)	94 (4.57)	−3.67%	0.57	0.002
**Treatment failure 2 (danger sign, hospital admission, death, malaria, pneumonia, diarrhoea, or reported fever ≥3 days)**					
Per-protocol	12 (0.60)	25 (1.28)	−0.68%	0.43	<0.001
Intention-to-treat	12 (0.56)	29 (1.41)	−0.847%	0.18	<0.001
**Treatment failure 3 (danger sign, hospital admission, death, malaria, pneumonia, diarrhoea, or measured fever [≥37.5°C])**					
Per-protocol	10 (0.50)	14 (0.72)	−0.22%	0.42	<0.001
Intention-to-treat	10 (0.47)	17 (0.83)	−0.36%	0.27	<0.001
**Treatment failure 4 (danger sign, hospital admission, death, malaria, pneumonia, or diarrhoea)**					
Per-protocol	7 (0.35)	3 (0.15)	0.20%	0.55	<0.001
Intention-to-treat	7 (0.33)	6 (0.29)	0.03%	0.36	<0.001

All 106 children who had treatment failure at the 1-week follow-up visit were visited at 2 weeks, at which time point all had fully recovered. In all, 3,922 (99.4%) of the enrolled children were followed up at 4 weeks for the vital status check. There were no deaths during the 4-week follow-up period.

Out of the 106 children with treatment failure at 1 week, 78 (73.6%) were from 1 cluster in the universal follow-up arm. In this cluster, the cases all occurred during a 2-month period (February and March 2016), and mainly comprised children who were reported still febrile at 1 week by their caregivers. Only 3 of these had a measured temperature of ≥37.5°C. A sensitivity analysis was done excluding this cluster; this analysis showed no difference in treatment failure between arms, with a risk difference of −0.27% to 0.20% (upper 95% CI 0.57–0.77) in the per-protocol population and −0.4% to 0.04% (0.39–0.76) in the intention-to-treat population.

In the per-protocol population, only 114 (5.7%) children in the conditional follow-up arm returned to the HEW after enrolment, 62 (54.4%) of them because the child was still sick on day 3 or had deteriorated. Only 3 did not recover by the time of the 1 week follow-up, and were therefore defined as treatment failures. Of the children who had treatment failure at 1 week, 91.1% (82/90) and 6.3% (1/16) had previously returned to the HEW in the universal and conditional follow-up arms, respectively. In the intention-to-treat cohort, 155 (7.3%) of the children in the conditional follow-up arm returned to the HEW, 66 (42.6%) because the child was still sick or deteriorating. Of the children who had treatment failure at the 1-week follow-up visit, 91.5% (86/94) and 15.8% (3/19) had previously returned to the HEW in the universal and conditional follow-up arms, respectively. Hence, most children who had treatment failure at day 8 in the conditional follow-up arm were not seen again by the HEW after enrolment, and only 3 children across both arms were taken to another provider. Few children subsequently sought care from another provider after having initially been seen by the HEW: 3.0% (59/1,993) in the conditional follow-up arm and 1.1% (22/1,953) in the universal follow-up arm, on average 3.2 and 3.4 days later, respectively, with no significant difference between arms (risk difference 1.79%, 95% CI −1.23, 4.82, *p* = 0.244). There was no difference between arms in time or money spent: the mean travel time to the other provider was 2.2 hours (95% CI 0.01, 5.3) in the conditional follow-up arm and 2.6 hours (95% CI 0.02, 4.5) in the universal follow-up arm (*p =* 0.82), and the mean cost for the efforts to seek care after visiting the HEW was 26.5 birr (US$1.12) (95% CI 7.8, 45.2) and 22.8 birr (US$0.96) (95% CI 15.6, 30.0) (*p =* 0.69), respectively. Of those who sought care from another provider after having been seen by the HEW, 88.9% went to a health centre, 6.2% to a hospital, and 3.7% to a private clinic. Few children received additional treatment; 3.2% (63/1,993) in the conditional follow-up arm and 1.5% (29/1,953) in the universal follow-up arm (*p* = 0.253). Based on examination of prescription notes/medicine packs or the caregivers’ report, 26 children received antibiotics (28.3%), 10 (10.9%) received antimalarials, and 8 (8.7%) received oral rehydration therapy and/or zinc. Only 1 child in each arm was admitted to hospital.

## Discussion

Conditional follow-up of children with non-severe unclassified fever in a low malaria transmission setting in Ethiopia was found to be non-inferior to universal follow-up through 1 week, with an average 2.7% of children across both arms having treatment failure at day 8. No deaths were recorded. While iCCM guidelines recommend universal follow-up for all children, regardless of symptom resolution, IMNCI guidelines recommend less intense conditional follow-up after 2 or 3 days (depending on malaria endemicity). To our knowledge, this study and a sister study in DRC [24] are the first to provide evidence that conditional follow-up is no less safe or marginally less safe than universal follow-up in children aged 2–59 months seen by CHWs.

Reported fever is one of the most common presenting symptoms of paediatric illnesses; fever incidence is variable, with country-specific reports from Africa showing a mean of 5.9 fever episodes annually per child under age 5 years [27]. Fever in children signifies systemic inflammation, typically in response to a viral, bacterial, parasitic, or, less commonly, non-infectious aetiology [28]. A number of studies have been conducted to establish the specific cause of fever in children who test negative for malaria, with the vast majority of fevers caused by viruses [8,9]. Present guidelines are based on clinical features that are unfortunately poorly predictive of the diseases causing fever; hence, low-cost, accurate, point-of-care diagnostics are needed to determine which children can benefit from antimicrobial treatment [29].

Several studies from sub-Saharan Africa provide convincing evidence that mRDT-negative febrile children can be safely managed without antimalarial treatment [12,30–33]. While the overall treatment failure rate in our study (2.7%) is similar to the low rate observed in a study of malaria-negative febrile children in 2 sites in Tanzania (3%) [12], it was significantly lower than the rate observed in Zambia (9.3%) [25] or in our sister study in DRC (10.1%) [24]. One explanation may be that the relatively lower malaria endemicity in Ethiopia leads to lower numbers of false-negative cases; parasite prevalence in children under 5 years in Ethiopia is 0.6% [34] versus 10%–75% in children aged 2–9 years in Zambia [35] and 22.6% in children under 5 years in DRC [36]. However, the 14% and 40% of children positive for malaria in the 2 study sites in Tanzania [12] suggest that factors other than malaria prevalence may also play a role.

With a steady decline in malaria transmission, the role and management of unclassified fevers will become more important [37,38]. With the epidemiology and burden of paediatric febrile illness shifting, understanding the aetiology of unclassified fevers in each context, and in particular in high-burden countries, is an important next step to improve management of these cases [28].

The communication between health providers and patients about the purpose and result of mRDTs is often poor [39,40]. Furthermore, primary healthcare workers often have low confidence in managing children with fever symptoms but negative tests for malaria. Recent studies show that mRDT-negative patients with cough or difficult breathing complaints in Malawi had 16.8 times higher odds of antibiotic overtreatment than mRDT-positive patients [41]; in Burkina Faso and Uganda both community and health facility workers prescribed antimalarials to mRDT-negative patients if no other fever cause was identified, often due to parental pressure [41,42]. However, when clear case management instructions were provided, as in this study, and non-malaria fever was introduced as a diagnostic term, HEWs felt empowered to withhold medicines, while simultaneously reassuring caregivers that their child was cared for [22]. This finding is supported by the low number of children in our study who were taken to another provider and provided with secondary treatment after being enrolled by the HEW.

The robust randomised controlled trial design is a particular strength of this study. Compliance both among HEWs, who gave the follow-up advice, and among caregivers, who followed the advice, was very high, indicating that these follow-up recommendations can be easily applied to routine case management practice. Yet, in an implementation setting, it is crucial that HEWs are well trained on counselling caregivers on when to come back for follow-up, as the high adherence seen in the controlled study setting may not transfer to routine practice.

A limitation of this study is that it did not collect sufficient clinical data on children at enrolment to be able to generate an understanding of which other symptoms or possible diagnoses were present. However, almost all children were followed up until 4 weeks, and none of them died or were referred between 1 week and four weeks, indicating that no child deteriorated to a severe condition. We were not able to further investigate fever aetiology among the children who had not recovered by 1 week, and we can therefore not speculate about the possible causes for these treatment failures. However, it is unlikely that this will have affected our results, as our failure outcome was purposefully designed to include any cause for failure, and was balanced between arms. Thus, we feel comfortable arguing that if children with potentially severe illness are excluded based on the presence of danger signs, the rest can be safely managed without a requisite visit on day 3.

In conclusion, we recommend that IMNCI guidelines in Ethiopia, which stipulate conditional follow-up of children with unclassified fever, remain unchanged, as our study demonstrated the safety of this approach in comparison to universal follow-up of similar children. Allowing CHWs to advise caregivers to bring children back only in case of continued symptoms might be a more efficient use of resources in these settings.

## Supporting information

S1 CONSORT Checklist Checklist for cluster-randomised controlled trials. (DOCX)

S1 Data Original dataset for enrolment and follow-up of enrolled children. (XLSX)

S1 Table Questionniare form used by HEWs to enrol children into the study. (XLSX)

S2 Table Questionniare form used by IEs to assess children at follow-up. (XLSX)

S1 Text Participant consent form for caregivers. (DOCX)

S2 Text Participant information sheet for caregivers. (DOCX)

## Abbreviations

CHW: community health worker
DRC: Democratic Republic of the Congo
HEW: health extension worker
iCCM: integrated community case management
IE: independent evaluator
IMNCI: Integrated Management of Neonatal and Childhood Illness
mRDT: malaria rapid diagnostic test
SNNPR: Southern Nations, Nationalities, and Peoples’ Region
