# Supplementary material for: Universal versus conditional day 3 follow-up for children with non-severe unclassified fever at the community level in Ethiopia: A cluster-randomised non-inferiority trial
Source: PLoS Med. 2018 Apr 17;15(4):e1002553. doi: 10.1371/journal.pmed.1002553 (PMC5903591; doi:10.1371/journal.pmed.1002553)
Supplement: S1 Text — (DOCX) [file pmed.1002553.s005.docx]

**Participant Consent Form for Caregivers**

**Research study: Universal versus conditional three-day follow-up visit for children with unclassified fever at the community level**

1. I confirm that I have read and understood the information sheet dated on………………, explaining the above research project and I have had the chance to ask questions about the study.
2. I understand that agreeing for my child to take part in the study is voluntary. I am free to withdraw my consent at any time without giving any reason and without any negative consequences. I may choose not to answer particular questions which may be asked as part of the study.
3. I understand that my name and my child’s name will not be linked to the research materials and any information that could identify me or my child will be kept strictly confidential. I understand that my child will not be identified or identifiable in any report, publications or presentations that result from this research.
4. I agree for the data collected from my child to be used in future research.
5. I agree to take part in the above research project.
6. Contact number of researcher: …………………

| **Name of caregiver:** | **Date:** | **Signature/Thumb print:** |
| --- | --- | --- |
| **Name of person taking consent:** | **Date:** | **Signature/Thumb print:** |

*To be signed and dated in the presence of the participant.

A copy of the consent form should be given to the parent/ guardian.
