# Supplementary material for: Universal versus conditional day 3 follow-up for children with non-severe unclassified fever at the community level in Ethiopia: A cluster-randomised non-inferiority trial
Source: PLoS Med. 2018 Apr 17;15(4):e1002553. doi: 10.1371/journal.pmed.1002553 (PMC5903591; doi:10.1371/journal.pmed.1002553)
Supplement: S2 Text — (DOCX) [file pmed.1002553.s006.docx]

**Malaria Consortium TRAction study**

**Caregiver of child under five information sheet and consent form**

We would like you to help with a research study. This information sheet will tell you what the research involves. Please take your time reading it. It can be read out to you if you choose. Please ask questions and you can talk it over with others if you wish.

Overall, the study aims to improve how children under five years of age with fever are managed by health extension workers. We would like to test whether febrile children with no malaria, pneumonia, or diarrhoea can be more safely managed if they receive a scheduled repeat visit to the health extension worker.

**Why has my child been chosen for the study?**

All children under five who are seen by health extension workers in this area for treatment of fever with a negative rapid diagnostic test for malaria, and no signs of pneumonia or diarrhoea are being asked to take part in this study. We hope to that about 4,300 children under-five will take part.

**What happens if I agree to take part?**

Your child will be seen by the health extension worker and treated as he or she normally would be. Half of the children will be asked to return in 3 days for a repeat evaluation whether they are still sick or not, and the other half will be told to return only if the child is still sick. Whether your child takes part is your choice; you may choose not to take part or to stop at any time. You will continue to receive the **same** diagnostic tests and medicines as usual if you do or do not agree to take part. If you take part you will help us find out if it is best for all children to have a repeat visit with the community health worker.

**What are the benefits of taking part?**

There are no direct benefits to you or your child, but this study hopes to improve the care of children with fever in the future.

**What are the possible disadvantages and risks of taking part?**

There are **no added risks** to taking part in this study. You may bring your child back to the health extension worker at any time if you feel that he or she is getting worse. In both study arms, and you will be asked to come back on day 3 if he or she has not improved, and a research team will check on your child’s status over the next 28 days. Whether you agree or not to take part **you will continue to receive** the same medicines and tests as usual.

**Will my participation in the study be kept confidential?**

Yes. The information will be stored by a number, not by your child’s name and not by your name. The information will **only** be available to the researchers working on the study.

**What will happen to the results of the study?**

They will be used to improve the management of children under-five with fever. The results will also be published in medical journals. You and your child will not be identified or identifiable in any reports of publications.

**What happens if the research study stops earlier than expected?**

If it does, we will provide you with clear information as to why.

**Who is doing and paying for the research?**

Malaria Consortium is carrying out the study, with funding from USAID through TRAction.

**If you have any questions at any time, please ask a member of the research team or you can contact:** The Project Manager, TRAction Project, Malaria Consortium, Ethiopia, Ayalkibet Abebe at 0911783433/0910690254 or you can contact sub country coordinator Esey Batisso 0916827686
